# No improvement in AUDIT-C screening and brief intervention rates among wait-list controls following support of Aboriginal Community Controlled Health Services: evidence from a cluster randomised trial

**DOI:** 10.1186/s12913-024-11214-6

**Published:** 2024-07-15

**Authors:** James H. Conigrave, K. S. Kylie Lee, Timothy Dobbins, Scott Wilson, José Padarian, Rowena Ivers, Kirsten Morley, Paul S. Haber, Julia Vnuk, Kushani Marshall, Kate Conigrave

**Affiliations:** 1 Faculty of Medicine and Health, Central Clinical School, University of Sydney, Sydney, NSW Australia; 2 Centre of Research Excellence in Indigenous Health and Alcohol, University of Sydney, Sydney, NSW Australia; 3 Centre for Alcohol Policy Research, La Trobe University, Melbourne, VIC Australia; 4 Institute for Positive Psychology and Education, Australian Catholic University, Sydney, NSW Australia; 5 The Edith Collins Centre (Translational Research in Alcohol, Drugs and Toxicology), Sydney Local Health District, Sydney, NSW Australia; 6 Faculty of Health Sciences, National Drug Research Institute, Curtin University, Perth, WA Australia; 7 Burnet Institute, Melbourne, VIC Australia; 8 School of Population Health, UNSW Sydney, Sydney, NSW Australia; 9 Aboriginal Drug and Alcohol Council of South Australia, Adelaide, South Australia Australia; 10 Sydney Institute of Agriculture and School of Life and Environmental Sciences, The University of Sydney, Sydney, NSW Australia; 11 Graduate School of Medicine, University of Wollongong, Wollongong, NSW Australia; 12 Drug Health Services, Royal Prince Alfred Hospital, Sydney, NSW Australia; 13 Aboriginal Health Council of South Australia, Adelaide, South Australia Australia; 14 Adelaide Rural Clinical School, The University of Adelaide, Adelaide, South Australia Australia; 15 School of Medicine, The University of Notre Dame, Sydney, NSW Australia

**Keywords:** Implementation research, Remote support, Alcohol screening, AUDIT-C, Training, Aboriginal australians

## Abstract

**Background:**

While Aboriginal and Torres Strait Islander Australians are less likely to drink any alcohol than other Australians, those who drink are more likely to experience adverse alcohol-related health consequences. In a previous study, providing Aboriginal Community Controlled Health Services (ACCHSs) with training and support increased the odds of clients receiving AUDIT-C alcohol screening. A follow-up study found that these results were maintained for at least two years, but there was large variability in the effectiveness of the intervention between services. In this study, we use services that previously received support as a comparison group to test whether training and support can improve alcohol screening and brief intervention rates among wait-list control ACCHSs.

**Methods:**

Design: Cluster randomised trial using routinely collected health data. Setting: Australia. Cases: Twenty-two ACCHSs that see at least 1000 clients a year and use Communicare as their practice management software. Intervention and comparator: After initiating support, we compare changes in screening and brief intervention between wait-list control services and services that had previously received support. Measurement: Records of AUDIT-C screening and brief intervention activity in routinely collected data.

**Results:**

During the reference period we observed 357,257 instances where one of 74,568 clients attended services at least once during a two-monthly data extraction period. Following the start of support, the odds of screening (OR = 0.94 [95% CI 0.67, 1.32], *p* = 0.74, $$B{F}_{10}$$$$\approx$$ 0.002) and brief intervention (OR = 1.43 [95% CI 0.69, 2.95], *p* = 0.34, $$B{F}_{10}$$$$\approx$$ 0.002) did not improve for the wait-list control group, relative to comparison services.

**Conclusions:**

We did not replicate the finding that support and training improves AUDIT-C screening rates with wait-list control data. The benefits of support are likely context dependent. Coincidental policy changes may have sensitised services to the effects of support in the earlier phase of the study. Then the COVID-19 pandemic may have made services less open to change in this latest phase. Future efforts could include practice software prompts to alcohol screening and brief intervention, which are less reliant on individual staff time or resources.

**Trial registration:**

Retrospectively registered on 2018-11-21: ACTRN12618001892202.

## Introduction

Aboriginal and Torres Strait Islander Australians are more likely to abstain from drinking alcohol than other Australians [1]. However, ongoing legacies stemming from colonisation, including trans-generational trauma, racism, and poor socioeconomic opportunities [2], have contributed to an increased prevalence of risky drinking (i.e., above national guidelines) [3]. Identifying at-risk individuals can help prevent or reduce harms by providing clients with support, brief interventions, or other treatments. Structured screening tools like AUDIT-C [4] can improve the detection of Aboriginal and Torres Strait Islander Australians at-risk from alcohol consumption [5]. However services may require training and support to implement AUDIT-C screening and alcohol brief intervention [6].

In Australia, there are many primary health services which are operated by Aboriginal and Torres Strait Islander Australian communities. Aboriginal Community Controlled Health Services (ACCHSs) provide comprehensive, evidence-based, preventive, and therapeutic health care [7, 8]. ACCHSs tailor their services to the needs of their respective local communities and offer culturally appropriate care which can help engage their clients [7]. As Aboriginal and Torres Strait Islander Australians are at greater risk of chronic diseases, ACCHSs often prioritise screening and preventive care [9]. Risky drinking is among the health risk factors that ACCHSs seek to address [10–12].

Screening for risky drinking can be particularly challenging in Aboriginal and Torres Strait Islander Australian contexts [13]. In some communities, disclosing at-risk alcohol consumption can be both embarrassing and stigmatising, making conversations with health professionals about drinking particularly stressful—especially when the health professional and client know each other [13].

Structured screening tools such as AUDIT-C can help guide conversations about drinking to ensure that critical information is collected to establish risk [5]. When clinicians in ACCHSs use AUDIT-C rather than unstructured drinking risk assessments, they identify more than three times as many clients at risk from drinking alcohol [5].

Regular screening with structured screening tools such as AUDIT-C is needed to support at-risk Aboriginal and Torres Strait Islander Australian clients in receiving timely treatment [5]. In a cluster-randomised trial, we found that training and support improved AUDIT-C screening at ACCHSs, yet did not boost rates of brief interventions. These improvements were sustained over 24 months [14, 15]. Despite these gains, we identified significant heterogeneity between services in both baseline screening rates and subsequent improvements. Services that received support initially had much lower baseline screening rates. This may have artificially inflated the intervention’s effect size, as these services had more room for improvement.

Such variability casts doubt on the generalisability of our findings, particularly for services with higher baseline screening rates. Given the large variability in AUDIT-C screening practices at ACCHSs, the effects of providing training and support may be context-dependent. To better understand the contexts in which support and training are beneficial, we must examine the program’s effects across various settings. We can accomplish this by comparing the impact of the support program on wait-list control services to services that previously received the intervention in our cluster-randomised trial.

In this study, we aim to replicate our previous finding that supporting ACCHSs improves the likelihood of clients being screened with AUDIT-C. We also test whether the program can increase the odds of clients receiving brief interventions^1^. To do so, we analyse routinely-collected data before and after wait-list control (“*Active Support*”) services received support. During this period the screening rates of services who previously received the support package (“*Previous Support*”) had stabilised, making them an appropriate comparison group. We examine if actively supporting services increases the odds of clients being screened with AUDIT-C (Hypothesis 1). We also test if active support improves the odds of clients receiving brief intervention (Hypothesis 2). By addressing these questions, we seek to clarify the effectiveness of supporting ACCHSs across differing contexts. This information is important for policy makers and researchers seeking to advocate for effective and appropriate programs that support at-risk Aboriginal and Torres Strait Islander Australians, ultimately informing efforts to improve outcomes for this population.

## Method

### Study design

This study is part of a larger wait-list controlled cluster randomised trial. Twenty two ACCHSs were randomised (equal allocation, stratified by remoteness) to receive training and support or to a wait-list control group. One- and two-year outcomes for Previous Support services relative to wait-list controls have previously been published [14, 15]. The primary outcomes were the odds of recording screening and of receiving brief intervention. The study design, research questions and analysis plan were retrospectively registered with the Australian New Zealand Clinical Trials Registry (ACTRN12618001892202), the study protocol has been published [6]. In this current study, we compared outcomes for the wait-list control group—now termed Active Support—before and after they received training and support, against outcomes for Previous Support services over the same timeframe.

**Fig. 1:**
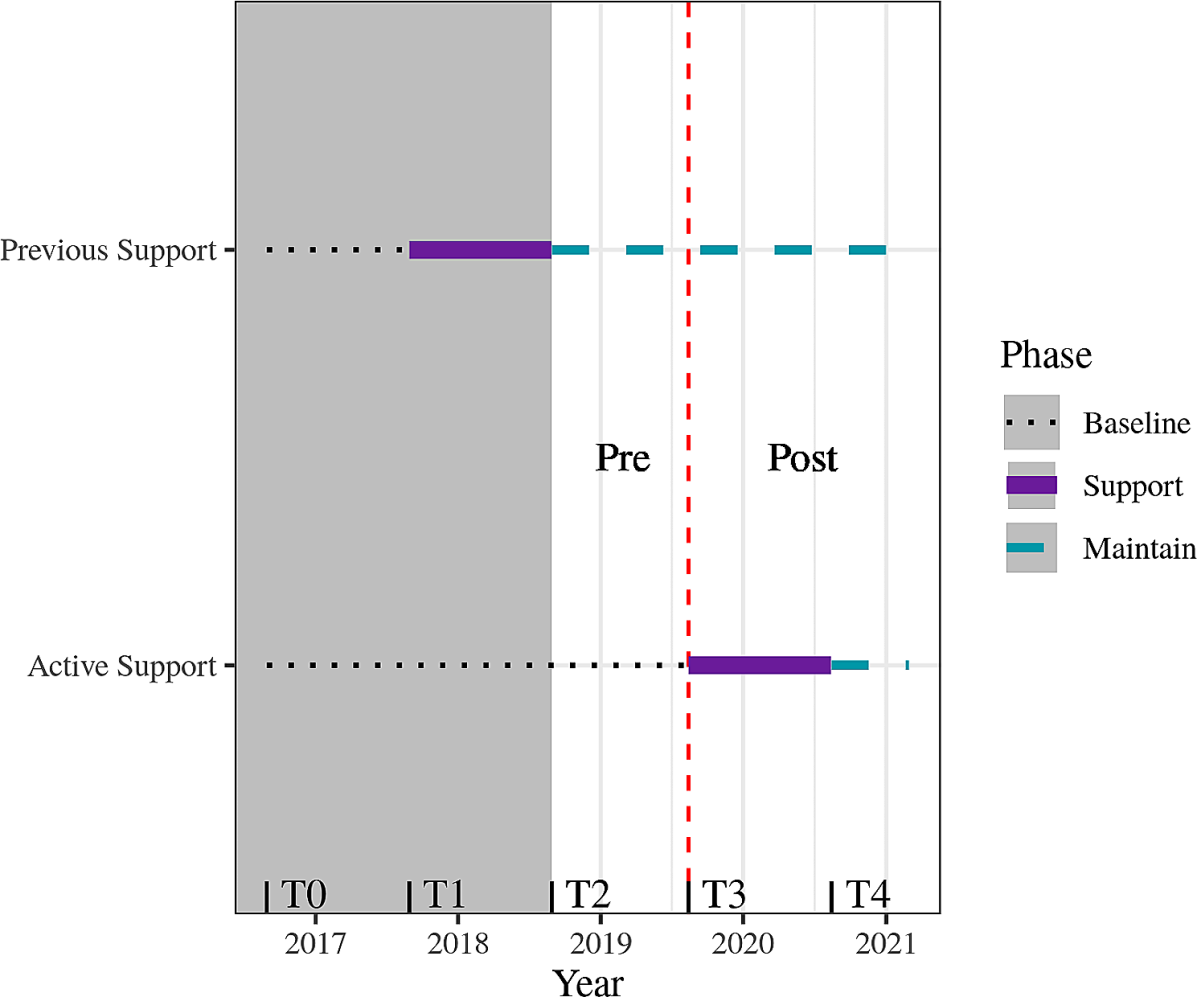
Diagram of study phases. The reference period for the current study is the white segment following the start of maintenance for Previous Support services. For this period, we test whether the odds of screening and brief intervention increased for Active Support services after the implementation of their support program (indicated by the dashed vertical red line) beyond any natural increase experienced by Previous Support services

Specifically, we tested whether Active Support increased the odds of clients being screened with AUDIT-C and receiving brief intervention. The broader trial had five time periods (Table 1). Both arms provided baseline data at ‘Time 0’. Previous Support services received support at ‘Time 1’ and then entered maintenance (receiving a lower level of support) at Time 2. Active Support services received support at Time 3 and then received a lower level of support until the end of Time 4. The reference period of the current study includes Time 2 through Time 4, that is, from 2018-08-29 to 2021-02-28 (Fig. 1). As Previous Support services received a consistent, low level of support over this period, they were used as a comparison group in the present study.

**Table 1:** Study phase start and end dates

Time	Stage	Date range
T0	Common baseline	2016 Aug-29 to 2017 Aug-28
T1	Early support	2017 Aug-29 to 2018 Aug-28
T2	Early maintenance	2018 Aug-29 to 2019 Aug-13
T3	Late support	2019 Aug-14 to 2020 Aug-13
T4	Late maintenance	2020 Aug-14 to 2021 Feb-28

### Involvement by Aboriginal and Torres Strait Islander Australians

Aboriginal Australians, including staff from two state-wide umbrella agencies for ACCHSs (in SA and NSW), were involved in formulating research questions, designing the study, and interpreting findings. Staff from participating ACCHSs helped to refine the study design and support program. Aboriginal Australian staff from the research team were key to recruiting services and implementing support.

### Recruitment

**Fig. 2:**
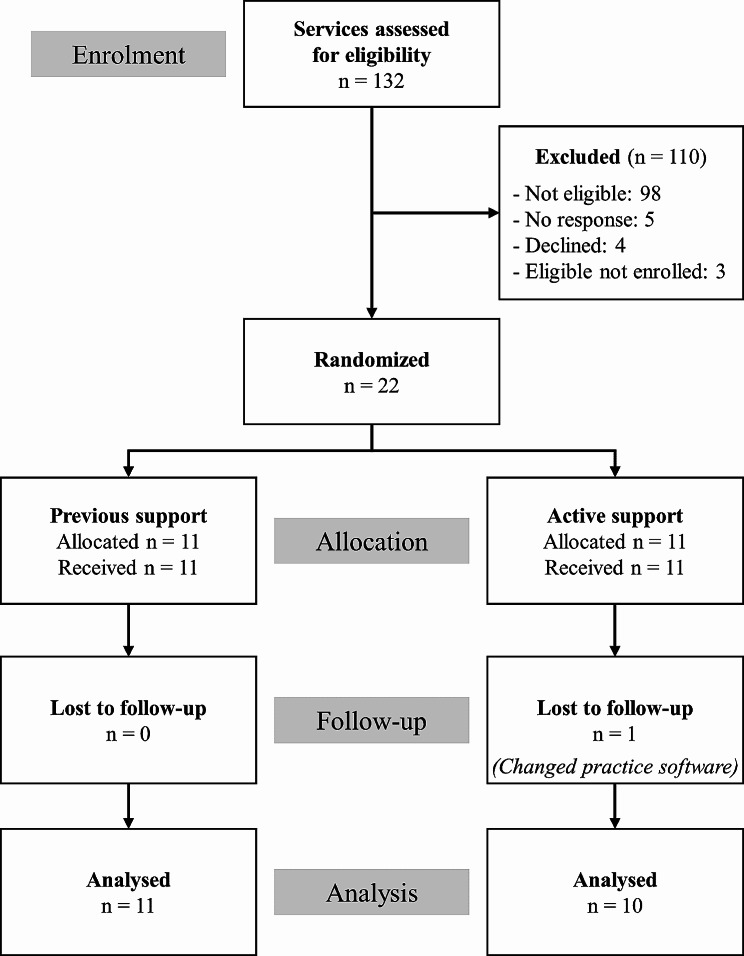
CONSORT diagram cluster trial extension

To be eligible, services had to be registered as ACCHSs, serve a minimum of 1000 Aboriginal or Torres Strait Islander Australian clients each year, and use Communicare as their practice management software. A power calculation was performed using PASS (‘Power Analysis & Sample Size’) [16]. Assuming that 60% of clients would be 16 years or older, that 57% would be screened for alcohol in a 12-month period, and an ICC of 0.04, we calculated that enrolling 10 Previous Support and 10 Active Support services would enable an increase in treatment provision of 13% to be detected with 80% power (two-sided significance, $\alpha$ = 0.05). Anticipating attrition, we recruited an additional service into each arm resulting in a total of 22 services (11 per arm). One Active Support service changed their practice management software during the trial, meaning they could no longer provide data and were excluded from the analyses in this paper (Fig. 2).

### Randomisation

We randomised based on remoteness strata (urban and inner regional; outer regional and remote; and very remote) using the 2011 standard Australian Geographical Classification System [17]. Services were randomised into the two trial arms by stratum. The randomisation process was performed in SAS by a researcher (TD) blinded to service identity. Services could not be blinded as to whether they were receiving support.

### Training and support provided

The details of the intervention have been described in detail elsewhere [6, 14, 15]. We provide a brief overview here. The intervention aimed to improve services’ capacity to identify and treat risky^2^ alcohol use. Service staff were provided with training on using AUDIT-C and delivering brief interventions or treatments when indicated. They received 2-monthly data feedback on alcohol screening and treatment at their service, along with resources, funding, and opportunities to network with peers to discuss challenges and successes. Where possible, support was tailored based on requests made by each service.

#### Service champions

Services nominated two representatives to promote engagement with the support program at their site. We encouraged services to nominate at least one clinician and at least one Aboriginal and/or Torres Strait Islander Australian health professional. Service champions received training at a national workshop and shared learnings at teleconferences held every second month.

#### National workshop

At the start of support, service champions attended a face-to-face, 2-day workshop. Presentations, group discussions, and role plays were given on topics such as how to screen with AUDIT-C, how to perform brief interventions, withdrawal management, relapse prevention medicines and how to engage families, carers, and communities on alcohol-related harms (including foetal alcohol spectrum disorders). Active Support services attended the 2-day workshop on 14–15 August, 2019.

### Telephone conferences

Telephone conferences, convened by an addiction medicine specialist (K.C.) and an Aboriginal research support officer, were held every 2 months with service champions. These conferences enabled champions to share learnings with staff from other services.

### Onsite training

Services were visited by an addiction medicine specialist (K.C.) and an Aboriginal Australian researcher or clinician. Content for on-site training (typically half-day) was adapted from that given at the national workshop based on staff interests and community needs. This training occurred within 5 months of the national workshop where possible^3^.

### Resources and funding

We gave services copies of the Australian alcohol treatment guidelines [18] and visual resources to use during brief interventions. We gave services funds to purchase further relevant resources. We gave Previous Support services AUD$9000 while Active Support services were provided with $3500—Active Support services received less funding as they were not asked to contribute as greatly to the design of the support program, and so had fewer demands placed on them.

### Practice management software support

Services received (optional) support from a research team member with both nursing skills and expertise in customising Communicare software.

### Data feedback

Services received an infographic PDF report on their services’ clinical activities related to alcohol consumption screening and management every two months. Reports included visualisations of their AUDIT-C screening rates, the proportion of their clients at risk from drinking and the numbers of clients who had received brief interventions or had been prescribed relapse prevention medicines.

### Online platform

An information repository and online forum was created for service champions to communicate with each other and to share resources.

### Maintenance (control condition)

Previous Support services (the comparison group) received maintenance support. This involved continued data feedback, access to the online platform, and (for the first 12 months of maintenance only) the option to attend a teleconference with other Previous Support services champions.

### Data analysis

Services provided us with routinely collected de-identified data from their practice software every two months. Data documented the clients who attended services in each period. Services also provided us with AUDIT-C screening results, and records of brief interventions, linked to client identifiers (IDs). We aggregated data so that rows summarised whether clients were given at least one AUDIT-C screening and at least one brief intervention during a given two-monthly data extraction period. As there were multiple extraction periods, the same clients appeared in the dataset multiple times. Additionally, clients were clustered by the services they attended. To manage these dependencies, we fit mixed-effects models using the R package ‘lme4’. Figures were produced with ggplot2 [19] and ggforce [20]. All confidence intervals are 95% Wald confidence intervals.

#### Fixed effects

To test whether support improved the odds of clients receiving AUDIT-C screening and the odds of brief intervention at Active Support services, we used multilevel logistic regression models. The two binary outcome variables of these models were whether clients were screened with AUDIT-C (primary outcome) or provided with a brief intervention within a two-month extraction period. The predictors were whether clients attended Active Support services, whether the observation occurred following the start of the implementation of the support package^4^, and whether the observation occurred following the start of the support package at an Active Support service (an interaction which tests the effect of the intervention).

These two models assess whether the implementation of the support package at Active Support services led to an increase in the likelihood of clients receiving AUDIT-C screening and brief interventions respectively.

#### Random effects

We included random intercepts for services and clients in the model predicting AUDIT-C screening. Due to convergence issues^5^, for the model predicting the odds of brief intervention we dropped the random intercept for clients and only included random intercepts for services. A random slope of the effect of time by service was included in both models.

#### Bayes factors

Bayes factors are indices of relative evidence of one model over another [21, 22]. Using the bayestestR package in R [21], we computed BIC-approximated Bayes factors to illustrate whether models which include the intervention effect (the interaction term) should be preferred to those without. A Bayes factor ($B{F}_{10}$) of 1 indicates that the data was equally likely under both models. A Bayes factor of 3 (or more) indicates that the hypothesised model should be preferred. A Bayes factor of 1/3 or less indicates that the null model should be preferred [21, 22].

## Results

Across the 2.50 year reference period (Aug 2018 to Feb 2021), 74,568 unique clients attended the 21 services. There were a total of 357,257 observations. Table 2 presents client and service features before (T2) and after (T4) Active Support services received training and support. While clients attending services in both conditions had similar characteristics, Previous Support services served more clients per year. Brief interventions were rarely recorded in either arms.

**Table 2:** Service and client features before and after wait-list controls received training and support

	Previous Support		Active Support	
	T2	T4	T2	T4
*Service characteristics*				
k	11	11	10	10
Average clients 000’s (SD)	3.0 (2.0)	2.4 (1.7)	1.6 (0.8)	1.4 (1.0)
Remoteness (n)				
Urban and inner regional	5	5	4	4
Outer regional and remote	2	2	3	3
Very remote	4	4	3	3
AUDIT-C screening rate (%)	16.0%	15.3%	12.6%	12.0%
Brief intervention rate (%)	0.4%	0.2%	0.1%	0.1%
*Client characteristics*				
n$^\dagger$	33,170	25,874	16,295	13,648
Observations per client	2.6 (1.6)	2.1 (1.1)	2.7 (1.6)	2.1 (1.1)
Age in years (SD)	38.0 (16.3)	39.5 (16.5)	38.3 (16.6)	39.0 (16.6)
Current drinkers	57.4%	56.7%	57.6%	57.8%
Mean AUDIT-C score (SD)	3.0 (3.4)	3.0 (3.4)	2.9 (3.3)	2.9 (3.3)

*Note* T2 = before wait-list controls received training and support, T4 = final year; k = number of services; n = number of clients. $\dagger$clients were identified using client IDs. It is likely that some clients attended multiple services, or that client ID was recorded incorrectly at times. Accordingly, the number of unique clients should be regarded as an estimate. One service dropped out of the Active Support arm as they changed their practice management software

### AUDIT-C screening

We tested whether training and active support improved the odds of clients being screened with AUDIT-C in each two-monthly extraction period relative to comparison services (Hypothesis 1) using a mixed-effects logistic regression model. We included a random slope for time by service and a random intercept for services and for clients. The odds of screening were predicted by ‘time’ (1 = after support implemented, 0 = pre-support), condition (1 = Active Support, 0 = Previous Support), and their interaction (the effect of attending a supported service, after the start of support; this term tests the effect of the intervention).

**Fig. 3:**
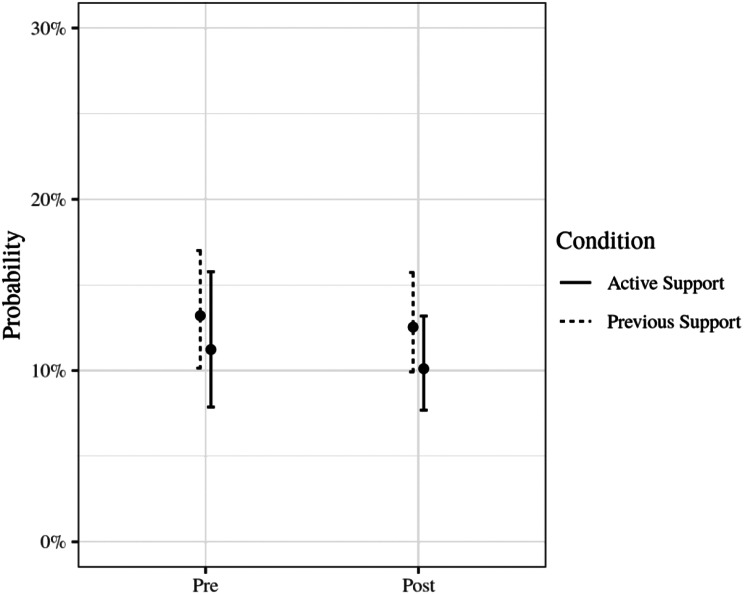
Predicted probability of a client being screened with AUDIT-C by study phase and condition

**Fig. 4:**
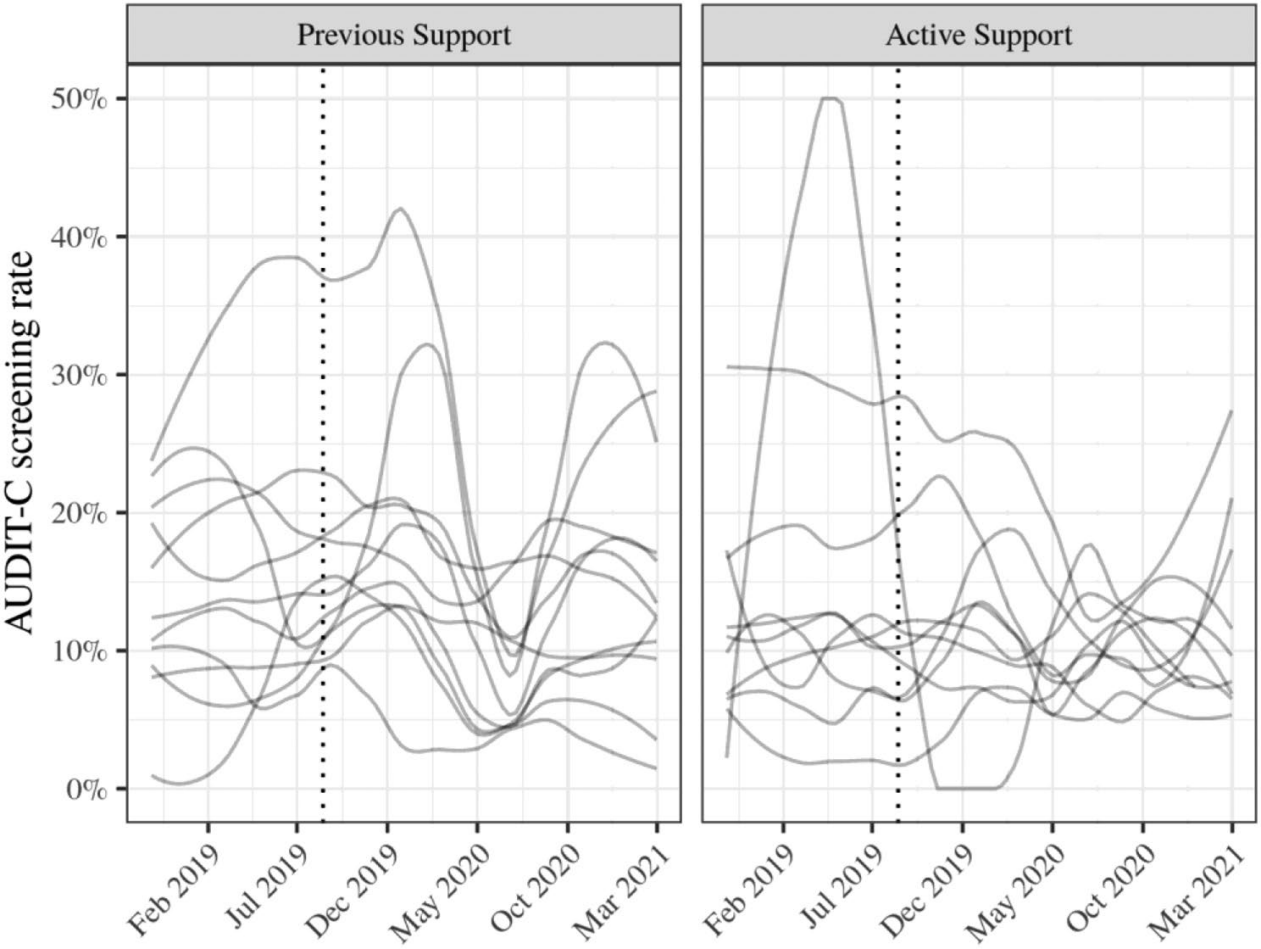
Smoothed AUDIT-C screening rates per service and extraction period

The regression results are presented in Table 3. The predictions of the model by condition and time are presented in Fig. 3. At T2—during Previous Support maintenance and before Active Support services received any support—the odds of recording AUDIT-C screening were low for Previous Support services, odds = 0.15 [95% CI 0.11, 0.20]. At baseline, the odds of AUDIT-C screening were not significantly different for clients attending Active Support services, relative to Previous Support services, OR = 0.83 [95% CI 0.52, 1.32]. The odds did not improve for Previous Support services following the start of support for Active Support control services, OR = 0.94 [95% CI 0.76, 1.17]. Similarly the odds did not change for Active Support following the start of their support, OR = 0.89 [95% CI 0.67, 1.17] (estimated from the model using the Delta method). The change in odds of AUDIT-C screening for Active Support services was not significantly different to the change for Previous Support services (the intervention effect; OR = 0.94 [95% CI 0.67, 1.32], *p* = 0.74, $$B{F}_{10}$$$$\approx$$ 0.002). That is, we do not have evidence that the support program improved AUDIT-C screening at Active Support services. AUDIT-C screening was clustered by service (ICC = 11.34%). Figure 4 demonstrates large variability in screening rates over time per service.

**Table 3:** Model predicting the odds of clients being screened with AUDIT-C by condition and study phase

Predictors	OR [95% CI]	lnOR	SE	$z$	p
*Fixed effects*					
Intercept	0.15 [0.11, 0.20]	-1.88	0.15	-12.38	< 0.001
Active Support	0.83 [0.52, 1.32]	-0.18	0.24	-0.78	0.43
Time	0.94 [0.76, 1.17]	-0.06	0.11	-0.53	0.60
Active Support & Time	0.94 [0.67, 1.32]	-0.06	0.17	-0.34	0.74
*Random effect summaries*					
${\tau }_{00}$id	0.07				
${\tau }_{00}$service	0.50				
${\tau }_{11}$service.Time	0.23				
${\rho }_{01}$service	-0.69				
ICC	11.34%				

*Note* lnOR = Natural logarithm of the OR (logits); SE = Standard error of the estimate (lnOR); $z$ = the ratio of the lnOR to its associated standard error; p values were estimated using the two-tailed Wald z-test. Active Support = the relative effect of attending an Active Support service, Time = Observation occurred following the start of active support. The interaction between Active Support and Time tests the effect of the intervention. Variable type of random effect summaries listed in rows. $\tau$ = random effect variance (lnOR). $\rho$ = correlation between random intercepts and slopes. ICC = conditional intraclass correlation coefficient. Analysis by original assigned groups

### Brief intervention

**Fig. 5:**
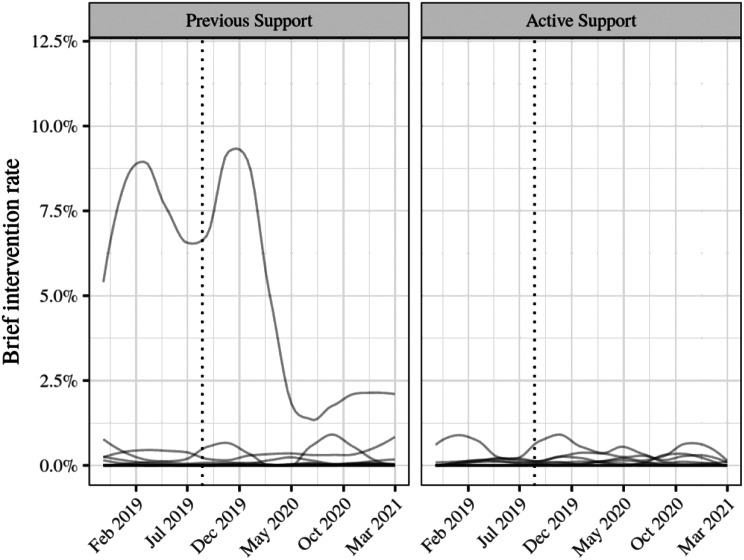
Smoothed brief intervention rates per service and extraction period

We tested whether training and support improved the odds of clients receiving a brief intervention in each two-monthly extraction period relative to comparison services (Hypothesis 2) using a mixed-effects logistic regression model. Random intercepts for services were included (the more complex variant of this model which also included random intercepts for clients did not converge and so was rejected). We included a random slope for time by service. The odds of recording a brief intervention were predicted by ‘time’ (1 = after support implemented, 0 = pre-support), condition (1 = Active Support, 0 = Previous Support), and their interaction (the effect of attending a supported service, after the start of the support; this term tests the effect of the intervention). Figure 5 demonstrates that only one (Previous Support) service showed a (six-month) period of regular brief interventions.

**Fig. 6:**
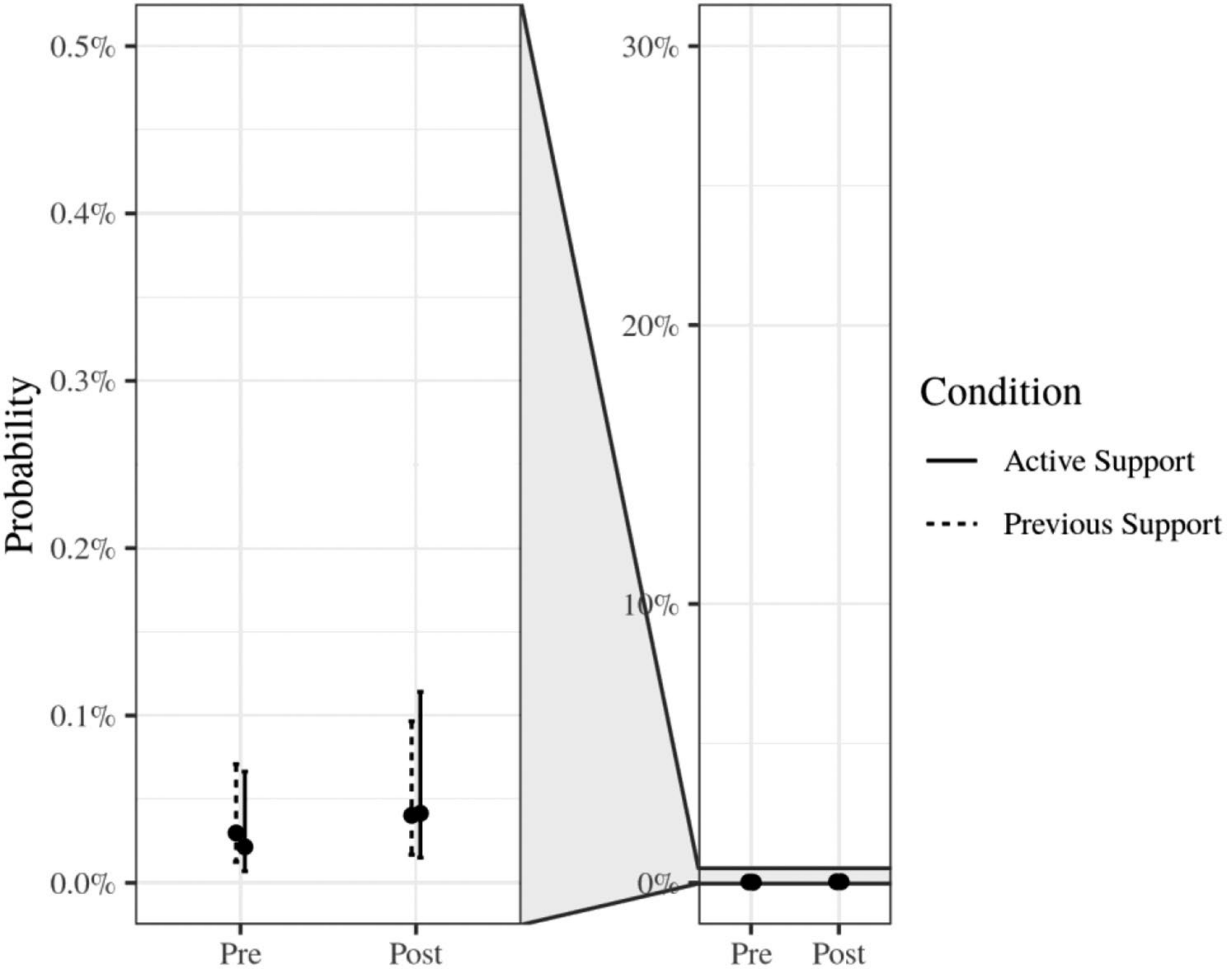
Predicted probability of a recorded brief intervention by study phase and condition. Brief interventions were rarely recorded. The left facet is a zoomed in view of the facet on the right

The regression results are presented in Table 4. The predictions of the model by condition and time are presented in Fig. 6. Prior to the implementation of support (T2) the odds of recording a brief intervention were extremely low for Previous Support services, odds = 0.00 [95% CI 0.00, 0.00] (ln-odds = -8.12). The odds were similar at baseline for clients attending Active Support services OR = 0.72 [95% CI 0.24, 2.17]. The odds did not improve for Previous Support services following the start of late-support, OR = 1.35 [95% CI 0.80, 2.28]. Similarly the odds did not change for Active Support services, OR = 1.93 [95% CI 1.00, 3.72] (estimated from the model using the delta method). The change for Active Support services after the implementation of their support was not significantly different from Previous Support services over the same period (the intervention effect; OR = 1.43 [95% CI 0.69, 2.95], *p* = 0.34, $$B{F}_{10}$$$$\approx$$ 0.002). That is, we did not find evidence that the support program increased the odds of clients receiving brief intervention at Active Support services. The odds of brief interventions occurring was highly clustered by service ICC = 58.76%. There were large standard errors for each term (and accordingly wide confidence intervals), which underscores the large degree of uncertainty in the model.

**Table 4:** Model predicting the odds of clients receiving brief intervention by condition and study phase

Predictors	OR [95% CI]	lnOR	SE	$z$	*p*
*Fixed effects*					
Intercept	0.00 [0.00, 0.00]	-8.12	0.44	-18.35	< 0.001
Active Support	0.72 [0.24, 2.17]	-0.33	0.56	-0.58	0.56
Time	1.35 [0.80, 2.28]	0.30	0.27	1.13	0.26
Active Support & Time	1.43 [0.69, 2.95]	0.36	0.37	0.96	0.34
*Random effect summaries*					
${\tau }_{00}$service	5.87				
${\tau }_{11}$service.Time	0.47				
${\rho }_{01}$service	-0.67				
ICC	58.76%				

*Note* lnOR = Natural logarithm of the OR (logits); SE = Standard error of the estimate (lnOR); $z$ = the ratio of the lnOR to its associated standard error; p values were estimated using the two-tailed Wald z-test. Active Support = the relative effect of attending an Active Support service, Time = Observation occurred following the start of active support. The interaction between Active Support and Time tests the effect of the intervention. Variable type of random effect summaries listed in rows. $\tau$ = random effect variance (lnOR). $\rho$ = correlation between random intercepts and slopes. ICC = conditional intraclass correlation coefficient. Analysis by original assigned groups

## Discussion

We previously found that providing Aboriginal Community Controlled Health Services (ACCHSs) with training and collaborative support improved AUDIT-C screening rates [14, 15]. In this paper we aimed to replicate this finding by examining whether waiting-list control services also saw improvements in AUDIT-C screening after they received that support package. We found that providing waiting-list control services with training and support did not improve screening rates, nor did it improve brief intervention rates. Our original finding might have been dependent on low baseline levels of AUDIT-C screening at Previous Support services and higher engagement from staff due to recent changes in AUDIT-C reporting requirements by the Australian Government. However, we were surprised that support did not tend to confer benefit to wait-list control services as it utilised many factors reflecting good practice. The failure of our finding to replicate might be due in part to the large differences between services and between communities, and to differing contextual factors—including the COVID-19 pandemic which coincided with provision of support for Active Support services. Due to these inconsistent findings, we expect that any benefits from training and supporting ACCHSs to improve AUDIT-C screening will be context-dependent.

Throughout our trial we identified great variability in service screening behaviour (within- and between services) [14, 15]. The original success of our support program may have been circumstantial (i.e., dependent on time-limited community needs, on policy environments, and/or on the presence of engaged and charismatic staff at services and within the research team). In our original analysis, which looked at the effects of support on Previous Support services, we found that services with high baseline screening tended to gain less from support [14]. Despite randomising services between our conditions during the common baseline (T0), wait-list controls tended to have higher baseline screening rates than Previous Support services—while all wait-list control services were using AUDIT-C for alcohol screening during the common baseline, three Previous Support services had never recorded an AUDIT-C screen. Accordingly, as wait-list control services were more likely to perform AUDIT-C screening at baseline, our original models would predict they would gain less from support. Staff at services with higher baseline screening might be more likely to disregard training if they perceive alcohol screening and treatment to already be appropriately prioritised at their services.

Changing conditions at participating services (inner setting), in the communities they serve, and across the health system (outer-settings) likely affected the implementation and effectiveness of our support package [23–25]. Near the start of support for Previous Support services, The Australian government introduced a requirement that ACCHSs report AUDIT-C screening [12] as part of their national key performance indicators. At this time we noted improvements in AUDIT-C screening rates for both experimental arms. This demand from the government is likely to have increased Previous Support services engagement with the support provided by the research team. This stimulus may have made it appear as though the support package was more effective than it was for Previous Support services and simultaneously may have reduced the amount of room for improvement among wait-list services.

Probably as a result of this new government reporting requirement, changes to the practice software also came about. At the time that Previous Support services received the intervention, Communicare did not routinely include AUDIT-C in the template for the ‘Adult Health Check’ (an annual government subsidised health check for Aboriginal and Torres Strait Islander people). Support given to some Previous Support services included the addition of AUDIT-C to Health Check templates. However by the time that wait-list controls received support, AUDIT-C had become a standard inclusion in the Adult Health Check in recent versions of the software. Again, this reduced room for improvement in wait-list control services.

Additionally, COVID-19 began spreading among the Australian population during the Active Support phase. Services understandably needed to prioritise pandemic control over preventative services during this period [26]. Further, restrictions on movement discouraged face-to-face service attendance [27, 28]. One Active Support service had to defer its onsite training due in part to COVID-related travel restrictions until after the end of the trial; however, other parts of the intervention were successfully delivered to this service. These changing circumstances speak to the complex environments in which ACCHSs operate. Ensuring support packages are useful and able to be tailored to diverse local contexts and staff requirements will be a challenge for future researchers and policy makers.

We found engaging wait-list services to be more difficult than Previous Support services. By the time we delivered the support program to waiting list control services, many of the original staff we liaised with during service recruitment (and who were eager to engage with the project) had changed employment—including several Chief Executive Officers. New staff members were typically unfamiliar with the study, and some did not see participation in the study as a priority for their service. Disruption caused by high staff turnover [29] at participating ACCHSs and competing concerns (including the early stages of the COVID-19 pandemic) are likely to have interfered with the capacity of staff to engage with the study intervention. High staff turnover has previously been linked with poorer screening and brief intervention program performance [30]. To help service staff stay engaged with support programs, support staff and service staff may need to have positive two-way, constructive working relationships [31]. Staff turnover necessarily disrupts such relationships.

Interventions which do not rely on the behaviour of individual service staff might be more consistent in their effects. For example, programs which change practice management software to include prompts to, or that makes screening and brief intervention easier to perform might naturally increase those activities without requiring staff to consciously change their practices [32]. Relying on already-strained staff resources to improve outcomes will likely give results which vary based on the motivations and time pressures of individual staff, service resources, and the extent to which services view change as a priority. While training may under some circumstances be a useful driver of practice-level change, its effects can be inconsistent and in some cases will not be a cost-effective use of resources. Training and support might help improve clinician confidence and awareness of the benefits of screening and brief intervention, but this may not be sufficient to change clinical behaviour [33].

Systematic study of facilitators and barriers to performing screening and brief intervention might reveal further opportunities to improve the effects of training and support. Perhaps the effects of training and support would become more consistent with greater investment. However, this might call into question the health economics of such an intervention. Ultimately, training and support might be less consequential than other drivers of clinician behaviour: clinical time pressures [30], competing clinical priorities [34], clinical software defaults [35], and health system policies and incentives.

### Strength and limitations

This study examined the effectiveness of providing training and support to 21 ACCHSs across multiple Australian states and territories. It used a ‘real-world’ routinely collected clinical data, rather than data collected under pressured experimental conditions. Accordingly, this study was designed well to test whether training and support can help increase screening and brief intervention at ACCHSs. However, there are limitations which reduce the extent to which our findings can be generalised.

Wait-list controls tend to receive less benefit than those who receive an intervention in a timely manner. Experimental research suggests that being on a waiting list to receive support can be taxing when one feels ready to change [36]. Staff who were originally enthusiastic about the program might have had less motivation after having to wait three years to receive support. If such a training and support program was adopted by policy makers, then recipient services would not have to wait such long periods for the support to start. This would likely result in improved engagement and outcomes.

We used Previous Support services as a comparison group. While this enabled us to examine outcomes for wait-list services, they are not a true control group as randomisation was not performed for this phase of the study. However, at this study’s baseline period, screening rates for both arms were similar (Table 2) meaning that these services likely do provide a fair comparison.

A limitation of our study design was that while we had core training elements, we adapted other elements of training and support based on participating services’ interests and needs. We chose this flexible structure as Aboriginal and Torres Strait Islander Australian communities have diverse needs and a one-size-fits-all approach can be inappropriate and unwelcome. While this may have made the intervention more likely to be beneficial, this flexibility makes comparisons between experimental arms difficult. Were differences in the effects of the intervention between the groups due to sampling error, or due to variation in how the intervention was tailored for each group based on the changing needs of services?

While this was a large scale study, we must be cautious in generalising our findings given the large heterogeneity between services. It is also possible that selection bias was introduced as services were recruited on a first-come basis. More motivated services may have been more likely to participate.

The low prevalence of brief interventions probably underestimates what was actually provided. The software ‘clinical item’ for recording brief intervention had to be actively searched for by clinicians. We suspect that busy clinicians may have had informal conversations about drinking with clients that were either not recorded, or were recorded using free-text, which was not captured in this study [37]. Modifying user interfaces to make it easy to record brief interventions following identification of at-risk alcohol consumption via AUIDT-C (e.g., with check-boxes) could encourage these activities and improve record keeping.

## Conclusion

We did not replicate the finding that providing ACCHSs with training and support can increase the odds of clients being screened with AUDIT-C. Additionally, consistent with our original study, we found that training and support do not increase the odds of recording that clients received brief interventions at ACCHSs. Factors which affect the outcomes of training and support might include the length of time since study recruitment, staff turnover, and changing service pressures due to COVID-19 restrictions. Needs for support vary between services and over time, and accordingly interventions aiming to support services will have inconsistent effects. Services might benefit more from training and support if their staff are unfamiliar with AUDIT-C, and baseline screening is low. Future intervention designs which are robust to changes in staffing and less reliant on sustained staff engagement may be more consistently beneficial.

## Data Availability

Data is not publicly available due to ethical mandates. Data is jointly owned by participating ACCHSs and Kate Conigrave. For enquiries, contact KC.

## Footnotes

1 previously we found no effect of the support program on the odds of clients receiving brief interventions.

2 above national guidelines.

3 For one service in the wait control arm, training was delayed and eventually provided via videolink after data collection ended due to circumstances beyond the control of the research team (including local factors and the COVID-19 pandemic).

4 whether a client visit occurred after 2019-08-15.

5 Issues with convergence often occur when models are too complex for the data they are trying to describe.
